# The Impact of Tranexamic Acid on Blood Loss Management in Primary Total Knee Arthroplasty: A Comprehensive Review

**DOI:** 10.7759/cureus.65386

**Published:** 2024-07-25

**Authors:** Sharad Sawant, Sanjay V Deshpande, Hitendra Wamborikar, Vivek H Jadawala, Anmol Suneja, Sachin Goel, Vatsal Patel

**Affiliations:** 1 Department of Orthopedics, Jawaharlal Nehru Medical College, Datta Meghe Institute of Higher Education and Research, Wardha, IND

**Keywords:** transfusion, antifibrinolytic, fibrinolysis, blood loss, total knee arthroplasty, tranexamic acid

## Abstract

Total knee arthroplasty (TKA) is a widely performed surgical procedure to restore function and relieve pain in patients with advanced knee arthritis. One of the key challenges in TKA is managing perioperative blood loss, which can lead to complications such as postoperative anemia and the need for blood transfusions. Tranexamic acid (TXA), an antifibrinolytic agent, has shown promising results in reducing blood loss and transfusion requirements in various surgical settings, including TKA. This comprehensive review synthesizes current evidence regarding the efficacy and safety profile of TXA in primary TKA. Mechanistically, TXA functions by inhibiting the breakdown of fibrin clots, promoting hemostasis, and minimizing blood loss. Clinical studies evaluating TXA in TKA have consistently demonstrated significant reductions in blood loss parameters, including total blood loss, postoperative drain output, and transfusion rates. Key findings highlight the efficacy of TXA across different dosing regimens and administration routes, with minimal associated risks of thromboembolic events or adverse effects. Comparative analyses with other blood conservation strategies underscore TXA's superiority in reducing transfusion requirements and its cost-effectiveness in clinical practice. The review also discusses current clinical guidelines and recommendations for TXA use in TKA, emphasizing optimal dosing strategies and patient selection criteria. Future research directions include exploring the long-term outcomes of TXA administration and its impact on functional recovery, and refining protocols to enhance its efficacy and safety further. In conclusion, TXA represents a valuable adjunct in blood loss management during primary TKA, offering substantial benefits in patient outcomes, healthcare resource utilization, and cost-effectiveness. Continued research efforts are warranted to optimize its use and expand its applicability in orthopedic surgery.

## Introduction and background

Total knee arthroplasty (TKA), commonly known as total knee replacement (TKR), is a surgical procedure to alleviate pain and restore function in patients suffering from advanced knee arthritis or other degenerative conditions. It involves replacing damaged knee joint surfaces with artificial components to improve mobility and quality of life [1]. Managing blood loss during TKA is crucial for several reasons. Excessive blood loss can lead to postoperative anemia, which may necessitate blood transfusions. These transfusions are associated with risks such as infection transmission, immunological reactions, and increased healthcare costs. Therefore, optimizing blood conservation strategies is pivotal in enhancing patient outcomes and reducing healthcare burden [2].

Tranexamic acid (TXA) has emerged as a valuable pharmacological agent in managing blood loss during TKA. TXA functions by inhibiting fibrinolysis, thereby reducing the breakdown of blood clots and minimizing blood loss. Its efficacy in various surgical settings, including orthopedic procedures like TKA, has been extensively studied and documented [3]. This comprehensive review aims to critically evaluate the impact of TXA on blood loss management in primary TKA. By synthesizing current evidence from clinical trials and studies, this review seeks to elucidate the efficacy, safety profile, optimal dosing strategies, and comparative effectiveness of TXA in reducing blood loss and transfusion requirements in TKA patients. Additionally, it aims to provide insights into clinical guidelines, recommendations, and future directions for research in this field.

## Review

Mechanism of action of tranexamic acid

How TXA Functions in Inhibiting Fibrinolysis

TXA works by inhibiting fibrinolysis through competitive bidding to the lysine-binding sites in plasminogen, thus preventing its activation into plasmin, the enzyme crucial for breaking down fibrin clots [4]. TXA mimics lysine at a molecular level, competing with fibrin for these binding sites on plasminogen. This competition effectively blocks plasminogen from interacting with fibrin, inhibiting plasmin formation and subsequent fibrin clot breakdown [4]. By occupying the lysine-binding sites on plasminogen, TXA maintains its closed conformation necessary for binding to fibrin, thereby preventing plasmin activation and stabilizing fibrin clots, thereby reducing bleeding [5]. Additionally, TXA suppresses the activity of plasminogen activators like tissue plasminogen activator and urokinase plasminogen activator, which further reinforces its antifibrinolytic effects [4]. The effectiveness of TXA in inhibiting fibrinolysis varies with dosage, as studies indicate a half-maximal inhibitory concentration of approximately 3.79 ± 0.17 mg/L under experimental conditions [4].

Pharmacokinetics and Pharmacodynamics

TXA displays a two-compartment pharmacokinetic profile after intravenous (IV) administration. It has a biological half-life of approximately two to three hours and is predominantly eliminated unchanged via glomerular filtration, with more than 94% of the dose excreted in the urine. Renal clearance of TXA is about 130-135 mL/min/1.73 m², indicating exclusive kidney elimination without significant tubular secretion or reabsorption. The oral bioavailability of TXA ranges from 34% to 47% compared to IV administration, and its absorption is unaffected by food intake. Intramuscular administration achieves therapeutic plasma concentrations (>15 mg/L) within 15 minutes, offering quicker onset compared to oral dosing [6]. TXA's primary pharmacodynamic effect involves inhibiting fibrinolysis by competitively blocking lysine binding sites on plasminogen, thereby preventing its conversion to plasmin. This antifibrinolytic action stabilizes fibrin clots, reducing blood loss, particularly in hyperfibrinolytic conditions. TXA may also exert anti-inflammatory effects by inhibiting the plasmin-mediated activation of complement, monocytes, and neutrophils. The consistent pharmacokinetic profile of TXA, along with its well-defined antifibrinolytic mechanism, underscores its effectiveness in managing excessive bleeding [4].

Efficacy of TXA

Review of Clinical Trials and Studies Evaluating TXA in TKA

The review of clinical trials and studies on TXA in TKA reveals several significant findings and recommendations. One key finding is that the timing of TXA administration does not significantly affect outcomes. A clinical trial involving 212 TKA patients compared TXA administration before surgical incision versus before tourniquet release and found no notable differences in postoperative bleeding or transfusion rates between the two timings. Additionally, administering a second dose of TXA three hours after surgery did not yield significant effects on outcomes [7]. Another important discovery is that combining TXA with local infiltration analgesia (LIA) can reduce perioperative blood loss. A study on 176 patients undergoing unicompartmental knee arthroplasty (UKA) demonstrated that TXA combined with LIA significantly decreased perioperative blood loss compared to a control group. Moreover, TXA+LIA group patients had shorter hospital stays, indicating potential improvements in recovery outcomes [8]. Intra-articular (IA) administration of TXA has also proven effective in TKA for reducing blood loss and transfusion rates. A study involving 202 patients undergoing primary TKA showed that IA TXA substantially reduced blood loss (average 1,220 vs. 1,900 mL, p < 0.001) and transfusion rates (0% vs. 24.75%, p < 0.001) compared to no TXA. This approach was effective and cost-effective, reducing the need for transfusions and associated costs [9]. The Veterans Affairs Pharmacy Benefits Management Services recommends using TXA at a standard dose of 10 mg/kg to minimize blood loss and transfusion requirements in TKA. Various protocols have demonstrated reduced total blood loss and transfusion rates compared to controls. However, TXA is contraindicated in cases of hypersensitivity, recent coronary or vascular stents, recent thromboembolic events, bleeding disorders, and hypercoagulable states. Topical TXA administration may be considered an alternative [10]. While TXA has shown efficacy in reducing blood loss and transfusion rates in TKA, the available evidence has some limitations. Meta-analyses support its benefits but underscore the need for further research to define optimal dosing regimens and ensure safety in high-risk patient groups. Therefore, TXA should be used judiciously, considering individual patient factors, particularly in higher risk populations [11]. Overall, current evidence supports TXA's role in TKA to mitigate blood loss and transfusion needs, with ongoing research to refine its application in clinical practice.

Comparison of Blood Loss Parameters With and Without TXA

TXA demonstrates significant efficacy in reducing postoperative blood loss and minimizing the need for blood transfusions in patients undergoing TKR. In one study, the TXA group exhibited a mean postoperative blood loss of 272.5 mL compared to 685 mL in the placebo group (p < 0.001). Total blood loss was also markedly lower in the TXA group (427.6 vs. 911.6 mL, p < 0.001). Additionally, fewer patients in the TXA group required blood transfusions compared to the placebo group [10]. In TKR patients, IA TXA administration similarly led to substantial reductions in blood loss (average 1,220 vs. 1,900 mL, p < 0.001) and transfusion rates (0% vs. 24.75%, p < 0.001) compared to no TXA. Combining IV and IA TXA further enhanced the effectiveness in reducing blood loss and transfusion risks [9]. In liposuction procedures, local administration of TXA significantly decreased blood loss from lipoaspirate. The average volume of blood loss in the TXA group was 130.8 ± 179 mL, representing a 61% reduction compared to 339.5 ± 384 mL in the control group. Moreover, the average volume of blood loss per liter of lipoaspirate was substantially lower in the TXA group (33.3 ± 24.7 mL) compared to the control group (65 ± 58 mL) [12].

Safety profile of TXA

Adverse Effects and Complications Associated With TXA

**Figure 1:**
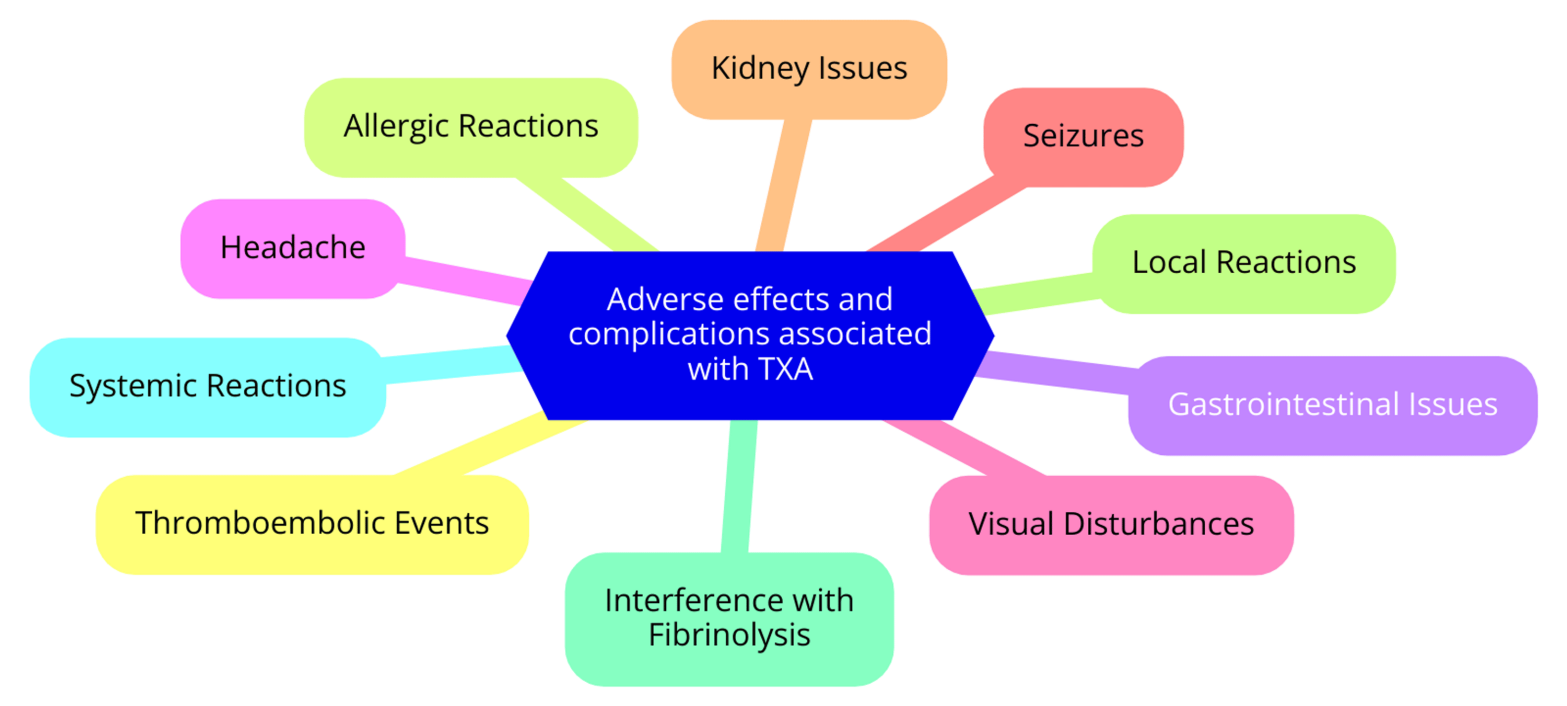
Adverse effects and complications associated with TXA TXA: tranexamic acid Image Credit: Sharad Sawant

TXA maintains a favorable safety profile when used in primary TKA. Numerous studies have consistently shown no significant differences in rates of thromboembolic events, wound complications, or other adverse effects between TXA and control groups in TKA patients. A systematic review and meta-analysis encompassing over 10,000 patients confirmed that TXA administration does not elevate the risk of venous thromboembolism or other adverse events following total joint arthroplasty [13]. Despite being an antifibrinolytic agent, TXA carries a minimal theoretical risk of increasing vascular events. Studies in contexts such as postpartum hemorrhage and acute traumatic injury have not indicated a heightened thrombotic risk associated with TXA use. TXA's superior safety profile compared to aminocaproic acid is attributed to its lower required daily dosage for efficacy [3]. Research on TXA administration during and after cesarean section underscored its ability to reduce bleeding without safety concerns significantly. In studies involving women with heavy menstrual bleeding, common adverse events associated with TXA included menstrual discomfort, headache, and back pain [14]. Potential adverse effects of TXA in TKA include allergic reactions, cardiovascular effects, gastrointestinal issues, and neurological symptoms. While rare cases of anaphylactic shock and allergic skin reactions have been reported, instances of hypotension and thromboembolic events do not appear to have significantly increased compared to placebo. Common side effects encompass nausea, vomiting, diarrhea, and abdominal pain. Rare cases may involve dizziness, seizures, or visual impairment. Overall, TXA's safety profile remains favorable, with no substantial rise in adverse events or complications compared to control groups in TKA patients, as evidenced by current research [15]. Adverse effects and complications associated with TXA are shown in Figure 1.

Risk-Benefit Analysis in TKA Patients

The risk-benefit analysis of TKA in older populations is multifaceted, encompassing both clinical outcomes and potential risks associated with the procedure. Studies have demonstrated that TKA can significantly benefit older patients in terms of clinical outcomes. For octogenarians, TKA has provided substantial pain relief, high satisfaction, and improved quality of life comparable to those seen in septuagenarians. Interestingly, octogenarians have reported higher mental and satisfaction scores postoperatively, indicating that TKA suits this age group when surgical indications are appropriate and risks are manageable. Similarly, nonagenarians have also benefited from TKA, experiencing significant pain relief and enhanced quality of life. Moreover, the procedure is cost-effective, delaying the need for nursing home placement and increasing the ability to receive home care [16].

Regarding functional outcomes, common comorbidities such as ischemic heart disease, atrial fibrillation, hypertension, and chronic obstructive airway disease did not significantly affect the outcomes of TKA. Older patients, including octogenarians and nonagenarians, showed improvements in physical function and pain relief after TKA despite these comorbidities [17]. However, the risk-benefit analysis must also consider the potential risks and complications associated with TKA, especially in the older population. Advanced age is a significant risk factor for mortality following TKA, with the risk of death within one year of the procedure increasing with age and higher mortality rates observed in patients over 80 years old. Comorbidities such as cerebrovascular disease, congestive heart failure, myocardial infarction, and renal disease are also associated with higher mortality rates after TKA [18]. Other complications, such as the need for blood transfusions, have been mitigated through TXA. Studies have shown that TXA can significantly reduce blood loss and transfusion requirements in TKA patients, thereby minimizing complications related to blood transfusions. Additionally, although TXA is an antifibrinolytic agent, studies have not found an increased risk of thromboembolic events with its use in TKA patients [19].

Optimal dosing strategies

Different Dosing Regimens Used in Clinical Practice

In vascular surgery, a typical dosing regimen for TXA involves an initial loading dose of 1 g, followed by a maintenance dose of 1 g every six to eight hours for two to five days. This regimen aims to sustain therapeutic levels of TXA throughout the perioperative period, potentially reducing blood loss and the need for transfusions [20]. Similarly, in orthopedic surgeries such as TKA, a common protocol includes a loading dose of 1 g and a maintenance dose of 1 g every six to eight hours for one to three days. This approach has effectively decreased blood loss and transfusion rates [21]. In cardiac surgery, a different dosing strategy is employed: a loading dose of 10 mg/kg, followed by a maintenance infusion of 1 mg/kg/hour until the end of the surgery. This regimen aims to maintain a consistent level of TXA throughout the surgical procedure, optimizing its hemostatic effects [22]. IV administration and repeated dose strategies are utilized for spine surgery. The IV regimen typically involves a preoperative bolus dose of 10-20 mg/kg, followed by a maintenance infusion of 1-10 mg/kg per hour throughout the surgery. Alternatively, a repeated dose administration strategy may include an initial bolus dose of 15 mg/kg, followed by an additional bolus three hours later [23]. In the context of postpartum hemorrhage, a fixed dose of 1 g of TXA in 10 mL (100 mg/mL) is administered intravenously at a rate of 1 mL per minute over 10 minutes. If bleeding continues after 30 minutes, a second dose of 1-g IV may be given [24]. For blunt trauma patients, a loading dose of 10 mg/kg is typically used, followed by a maintenance infusion of 1 mg/kg/hour until the end of surgery. This regimen aims to mitigate blood loss and improve outcomes in critically ill patients [3]. In complex spine procedures, a higher loading dose of 15 mg/kg is often administered, followed by a maintenance infusion of 100 mg per hour for up to five hours postoperatively. This approach ensures adequate TXA levels throughout the perioperative period, potentially reducing blood loss and transfusion requirements [25]. Overall, while the optimal dosing regimen remains unclear, general guidelines for TXA administration involve IV bolus doses ranging from 10 to 20 mg/kg preoperatively, followed by maintenance infusions of 1-10 mg/kg/hour throughout the surgery. Lower dose regimens, such as 10 mg/kg + 1 mg/kg/hour, 10 mg/kg + 2 mg/kg/hour, 15 mg/kg, and 15 mg/kg + 1 mg/kg/hour, have also been utilized in various studies. The choice of dosing regimen may depend on the specific surgical context and the individual patient's characteristics [23].

Factors Influencing Dosing Decisions

Factors influencing dosing decisions for TXA in primary TKA include patient characteristics, surgical techniques, and the specific route of TXA administration. One of the key factors is patient weight, as the optimal dose of TXA can vary accordingly. For IV administration, a dose of 30 mg/kg has proven effective, while for IA administration, a dose of 1-3 g is recommended. Although age may influence dosing decisions, there is no clear evidence suggesting that age significantly affects the efficacy or safety of TXA in TKA [26]. Patients with comorbidities, such as bleeding disorders or those on anticoagulant medications, require special consideration when administering TXA. These patients may need closer monitoring and potentially adjusted dosing. The type of surgery, bilateral or unilateral TKA, can also impact dosing decisions. There is no consensus on the optimal timing for single-dose TXA administration in bilateral TKA. Additionally, surgical techniques, such as the type of anesthesia or the extent of tissue dissection, can influence bleeding patterns and the subsequent need for TXA [27].

IV administration of TXA effectively reduces blood loss and transfusion rates, with an optimal dose range of 1-6 g and 30 mg/kg being a commonly used regimen. IA administration of TXA is similarly effective, with an optimal dose range of 1-3 g, and it has been shown to reduce blood loss and transfusion rates significantly. A combined IV and IA administration regimen has also effectively reduced blood loss and transfusion rates. Both low-dose (IV+IA injection of 1 g) and high-dose (IV+IA injection of 2 g) combined regimens show significant reductions compared to single-route administration [28]. TXA is safe and well-tolerated in TKA patients, with studies indicating no significant differences in the rates of thromboembolic events, wound complications, or other adverse effects between TXA and control groups. TXA effectively reduces blood loss and transfusion rates in TKA patients. Optimal dosing strategies are based on patient weight and the route of administration to achieve the desired efficacy and safety outcomes [29].

Impact on transfusion rates

Reduction in Transfusion Requirements With TXA

TXA has been extensively studied for its ability to reduce blood transfusion requirements in patients undergoing TKA. A meta-analysis by Huang et al. found that TXA reduced the number of blood transfusions per patient by 0.78 units and the volume of blood transfusion per patient by 205 mL. Total, intraoperative, and postoperative blood losses decreased by 408, 124, and 214 mL, respectively [30]. Another study by Pongcharoen and Ruetiwarangkoon found that the differences were insignificant, while TXA reduced the transfusion rate in patients undergoing UKA. However, the study did show that combining TXA with LIA significantly reduced the hemoglobin (Hb) drop compared to control groups [31]. A study by Evangelista et al. demonstrated that TXA reduced the transfusion rate from 15% to 1% in patients undergoing total hip arthroplasty (THA). The study also showed a significant reduction in blood loss and Hb drop in patients receiving TXA compared to those without [11]. Furthermore, a Cochrane Review found that TXA use was associated with a 38% reduction in the probability of transfusion, though it did not affect mortality rates. A single-center retrospective observational study found that TXA reduced the number of patients requiring blood transfusions from 41 in the non-TXA group to 7 in the TXA group. Overall, TXA has significantly reduced blood transfusion requirements in patients undergoing primary TKA, with various studies demonstrating reductions in transfusion rates and blood loss [32].

Cost-Effectiveness Considerations

The available evidence indicates that using TXA in primary TKA is highly cost-effective. Several studies have highlighted significant cost savings associated with reduced transfusion rates due to TXA use. For instance, a retrospective study demonstrated that IA TXA reduced the transfusion rate from 24.75% to 0%, leading to substantial cost savings. Additionally, another study reported that hospitalization costs were €374 higher in the control group compared to the TXA group for TKA patients [33]. Cost-effectiveness analyses further support the use of TXA in TKA. A randomized controlled trial meta-analysis found that IV and local administration of TXA significantly reduced hospitalization costs for TKA patients. A break-even analysis for reverse total shoulder arthroplasty indicated that routine use of TXA would be cost-effective if it prevented just one periprosthetic joint infection out of 10,583 procedures [34]. When comparing oral and IV administration of TXA, a systematic review and meta-analysis revealed that oral TXA had a significantly lower total mean cost ($75.41) than IV TXA ($580.83) in THA patients. This suggests oral TXA may offer an even more cost-effective option for TKA patients [35].

Comparison with other blood conservation techniques

TXA vs. Other Pharmacological Agents

Multiple studies have demonstrated that TXA, whether administered systemically or locally, significantly reduces total blood loss and transfusion rates in patients undergoing TKA compared to placebo. A meta-analysis found that TXA reduced total blood loss by an average of 435 mL and decreased the transfusion rate by 41% in TKA patients [10]. Aprotinin, another antifibrinolytic agent, has also proven effective in reducing perioperative blood loss and transfusion requirements in major orthopedic surgeries, including TKA. A randomized trial comparing aprotinin and TXA in cardiac bypass surgery found that aprotinin was more effective than TXA in reducing postoperative blood loss [36]. Similarly, epsilon-aminocaproic acid (EACA), another antifibrinolytic drug, has effectively reduced blood loss and transfusion needs in major orthopedic procedures. A randomized trial comparing EACA and aprotinin in cardiac bypass surgery found that EACA was non-inferior to aprotinin in reducing blood loss and fibrinolysis [36]. Overall, multiple studies have not identified significant differences in rates of thromboembolic events, wound complications, or other adverse effects between TXA and control groups. A comprehensive meta-analysis concluded that TXA does not increase the risk of venous thromboembolism or other adverse events in total joint arthroplasty patients. The safety profiles of aprotinin and EACA also appear generally favorable, with no major safety issues reported based on the available evidence [37].

TXA vs. Nonpharmacological Methods

The available evidence strongly supports the use of TXA in primary TKA. Numerous studies have confirmed TXA's efficacy in reducing blood loss and transfusion requirements. A meta-analysis of 41 randomized controlled trials found that TXA, whether administered systemically, topically, or in combination, significantly reduced total blood loss and transfusion rates compared to placebo [38]. Another meta-analysis revealed that TXA reduced total blood loss by an average of 435 mL and the transfusion rate by 41% in TKA patients [33]. The literature also supports the safety profile of TXA in this context. Several studies have not identified significant differences in the rates of thromboembolic events, wound complications, or other adverse effects between TXA and control groups [39]. A systematic review and meta-analysis involving over 10,000 patients concluded that TXA does not increase the risk of venous thromboembolism or other adverse events in total joint arthroplasty patients [40]. Both systemic administration and local (IA) administration of TXA have effectively reduced blood loss and transfusion rates in TKA. Systemic TXA, typically administered intravenously, has proven effective, while IA TXA offers the additional advantage of minimizing potential systemic side effects [41]. The optimal dosage of TXA is generally considered 1-2 g, as higher doses do not provide additional benefits and may increase the risk of adverse events [3]. Compared to other blood conservation techniques, TXA, whether administered systemically or locally, emerges as a safe and highly effective strategy for reducing blood loss and transfusion requirements in primary TKA. It potentially outperforms nonpharmacological techniques such as hypotensive anesthesia and cell salvage [10]. However, a multimodal approach that integrates various techniques may offer additional benefits.

Clinical Guidelines and Recommendations

TXA has proven to be an effective intervention for reducing blood loss and transfusion requirements in primary TKA. Multiple studies have shown that TXA can decrease postoperative blood loss by up to 60% compared to placebo. Additionally, TXA has been found to lower the need for blood transfusions by one-third to one-half in TKA patients. Importantly, TXA use has not been associated with an increased risk of thromboembolic complications or other adverse events compared to control groups. Moreover, the reduced blood loss and transfusion requirements with TXA contribute to cost savings for healthcare systems [38]. TXA can be administered via IV, topical/IA, or oral routes, and combinations of these methods have all been effective in reducing blood loss and transfusion needs in TKA compared to placebo. While optimal dosing is still under investigation, evidence supports using a single dose of IV TXA (10-20 mg/kg) or IA TXA (1-4 g) as effective strategies for managing blood loss in TKA. Additionally, administering IV TXA before the surgical incision may reduce blood loss and transfusion needs more than postincision administration [26].

## Conclusions

TXA represents a significant advancement in managing blood loss during primary TKA. Through its mechanism of action in inhibiting fibrinolysis, TXA has consistently demonstrated efficacy in reducing perioperative blood loss and transfusion requirements without compromising patient safety. The evidence synthesized in this review underscores the robustness of TXA as a blood conservation strategy in TKA, highlighting its potential to improve clinical outcomes and reduce healthcare costs. However, while TXA has shown clear benefits, ongoing research is needed to refine optimal dosing protocols, assess long-term outcomes, and further elucidate its role in specific patient populations. Future studies should explore novel administration techniques and comparative effectiveness with emerging blood conservation strategies. By enhancing our understanding and implementation of TXA in TKA, clinicians can optimize surgical outcomes and enhance patient care in orthopedic practice.
